# Clinical Genetic Testing of Periodic Fever Syndromes

**DOI:** 10.1155/2013/501305

**Published:** 2013-01-01

**Authors:** Annalisa Marcuzzi, Elisa Piscianz, Giulio Kleiner, Alberto Tommasini, Giovanni Maria Severini, Lorenzo Monasta, Sergio Crovella

**Affiliations:** ^1^Laboratory of Immunopathology, Institute for Maternal and Child Health (IRCCS) “Burlo Garofolo”, 34137 Trieste, Italy; ^2^Department of Medical Sciences, University of Trieste, 34100 Trieste, Italy

## Abstract

Periodic fever syndromes (PFSs) are a wide group of autoinflammatory diseases. Due to some clinical overlap between different PFSs, differential diagnosis can be a difficult challenge. Nowadays, there are no universally agreed recommendations for most PFSs, and near half of patients may remain without a genetic diagnosis even after performing multiple-gene analyses. Molecular analysis of periodic fevers' causative genes can improve patient quality of life by providing early and accurate diagnosis and allowing the administration of appropriate treatment. In this paper we focus our discussion on effective usefulness of genetic diagnosis of PFSs. The aim of this paper is to establish how much can the diagnostic system improve, in order to increase the success of PFS diagnosis. The mayor expectation in the near future will be addressed to the so-called next generation sequencing approach. Although the application of bioinformatics to high-throughput genetic analysis could allow the identification of complex genotypes, the complexity of this definition will hardly result in a clear contribution for the physician. In our opinion, however, to obtain the best from this new development a rule should always be kept well in mind: use genetics only to answer specific clinical questions.

## 1. Introduction

Periodic fever syndromes (PFSs) represent a wide group of diseases characterized by recurrent attacks of apparently unprovoked inflammation and are thus considered among the so-called autoinflammatory diseases.

For the clinician, the question whether a patient suffers from a PFS usually arises after the evaluation and exclusion of more common clinical problems associated with fevers and inflammation, such as chronic infections, systemic autoimmune diseases, and paraneoplastic inflammatory conditions [1]. However, the pattern of associated clinical manifestations, the age at disease onset and, above all, the stereotypic recurrence of attacks can induce the suspicion of a PFS. A key to diagnose PFSs appears to simply be the consideration of its evenience [2, 3]: this can diminish the delay in time to diagnosis, avoiding in some cases repeated invasive and unsuccessful investigations performed to exclude more common multifactorial disorders.

A definite diagnosis is made easier today thanks to improved feasibility of genetic analysis for most PFSs: Familial Mediterranean Fever (FMF), Mevalonate Kinase Deficiency (MKD), Tumor Necrosis Factor Receptor-Associated Periodic Syndrome (TRAPS), and Familial Cold Auto-inflammatory Syndromes (FCAS). Indeed, each of these diseases can be identified and diagnosed by the detection of mutations in specific genes (*MEFV*, *MVK*, *TFRSF1A*, and *NLRP3/NLRP12*, resp.) [4] (Table 1).

A genetic cause is not yet known for another PFS, called PFAPA (Periodic Fever, Aphthous stomatitis, Pharyngitis, and cervical Adenitis). This is a common and benign condition that can be easily diagnosed from clinical data, not requiring specialized laboratory investigations [5].

All these autoinflammatory diseases are characterized by recurrent flares of systemic inflammation, presenting sudden fever episodes associated with elevation of acute phase reactants and with a number of clinical manifestations that might include inflammation of serosal surfaces and joints, skin rashes of unknown origin, lymphadenopathy, arthritis, as well as the involvement of other organs such as muscles and the central nervous system [6]. Rheumatic manifestations are extremely common and highly variable in their presentation and course in PFSs. In most cases, however, the typical recurrence of attacks with symptoms-free intervals can help in differentiating the disorder from a chronic rheumatic disease. Furthermore, the pattern of musculoskeletal involvement and concomitant manifestations can help to formulate the correct diagnosis, which can be confirmed by genetic testing [1]. A family history of similar problems, when present, enforces the idea of a genetic origin of the disease.

Due to some clinical overlap between different PFSs, differential diagnosis can be a difficult challenge that can require sequential or simultaneous analysis of different genes. In fact, there are no universally agreed recommendations for most PFSs. Various attempts have been made in order to develop clinical criteria or guidelines to identify which of these patients should be considered for genetic testing [7, 8]. Databases such as INFEVERS (http://fmf.igh.cnrs.fr/ISSAID/infevers/) and the Eurofever Project (http://www.printo.it/eurofever/autoinflammatory_diseases.asp) have been developed to collect clinical and genetic information on patients with all monogenic autoinflammatory syndromes, including HPFs. This has led to improved recognition of the spectrum of clinical problems associated with these conditions as well as development of targeted treatment strategies [9].

In this paper, we focus our discussion on the effective usefulness of genetic diagnosis. Even if a molecular diagnosis is not always fundamental for the choice of an effective treatment, it is of great utility in the followup of patients, avoiding inadequate diagnostic procedures and focusing on the risk of specific complications, such as amyloidosis. The aim of this paper is to establish how much can the diagnostic system improve, in order to increase the success of PFS diagnosis.

## 2. Clinical Considerations and Treatment

Hereditary PFSs encompass a rare group of diseases that share lifelong recurrent episodes of inflammatory symptoms and an acute phase response. Periodic fevers are typically present in the pediatric population, but a disease onset in adult age is not a rare occurrence for some conditions [10].

This paper will focus on four hereditary PFSs, which include two autosomal recessive conditions, FMF and MKD, and a group of autosomal dominant diseases, including TRAPS and FCAS [11]. Moreover, in pediatrics, these pathologies have been distinguished from the more common and usually self-limiting PFAPA [11]. Other autoinflammatory syndromes, associated with fever, show a continuous inflammatory phenotype but not necessarily with a periodic feature. This category includes diseases such as MWS (Muckle-Wells Syndrome), CINCA (Chronic Infantile Neurologic Cutaneous and Arthritis) syndrome, PAPA (Pyogenic Arthritis, Pyoderma gangrenosum, and Acne), Behçet's disease, adult Still's disease, and SoJIA (Systemic-onset Juvenile Idiopathic Arthritis) syndrome.

Not all inherited PFSs are rare diseases and for most of them the prevalence can vary depending on the ethnology [8, 12]. As an example, FMF has a high prevalence, from 1 : 500 to 1 : 256 in different ethnic groups of non-Ashkenazi Jews [13], while MKD is a rare disease, and it is found predominantly in north-western Europe, with a carriage rate of MVK V377I of 1 : 350 [14]. Among the autosomal dominant PFSs, TRAPS is thought to be the most common form of dominant PFS in Europe [14].

The onset of symptoms occurs generally in childhood, usually in the first year of life for MKD and with more variability for other PFSs. Besides, the diagnosis of PFSs can be formulated later due to mild or unclear symptoms that overlap among different diseases and sometimes remain for long time misinterpreted or ignored [15]. In fact, the current approach for the diagnosis of PFSs in children may not be adequate for adults, in which the diseases are often underdiagnosed, and there is the need for specific diagnostic algorithms [16, 17].

The diagnosis of most periodic fevers in children is based on clinical history and physical examination. The management of the acute attacks is nonspecific and poorly standardized in PFSs. Similarly, long treatments are specifically designed to reduce the number and severity of the inflammatory attacks, as well as to prevent the development of amyloidosis.

### 2.1. Familial Mediterranean Fever (FMF)

The FMF (MIM 249100) is the most frequent hereditary recessive PFS [18]. It predominantly affects populations from Mediterranean descent in which the frequency of carriers is high [19, 20]. In the vast majority of patients FMF becomes clinically apparent before the age of 20. Signs of painful serositis accompanying fever are the hallmark of the disease [21].

Acute attacks are characterized by a high-grade fever and inflammation of one or more serous membranes (peritoneum in 90% of cases, pleura, synovial membrane, tunica vaginalis, and pericardium). Fever usually lasts less than three days and in most cases a single pick is observed. There are usually no symptoms between acute attacks, although the joint involvement and myalgia may persist for several weeks.

Colchicine is the first-choice treatment for FMF, but a small percentage (5–10%) of patients does not show a complete response [22, 23] to this drug and needs additional treatments such as interleukin-1beta (IL-1*β*) inhibitors [24].

### 2.2. Mevalonate Kinase Deficiency (MKD)

MKD (MIM 260920) is a PFS identified in 1984 [25]. The first clinical episode usually begins before the end of the first year of life. Attacks start with chills, followed by a sharp rise in body temperature accompanied by cervical lymphadenopathy and abdominal pain. Hepatomegaly, splenomegaly, arthralgia, skin rash, diarrhea, and vomiting are common symptoms [26]. Mean attack duration is 5–7 days. Intervals between attacks usually range from 4 to 8 weeks and tend to increase with age after adolescence.

Suspect of MKD is usually confirmed by elevated concentrations of mevalonic acid in urines during the flares and/or by a decreased enzymatic activity of mevalonate kinase during asymptomatic intervals. The diagnosis is consequently defined by genetic testing of the *MVK* gene. A marked elevation of polyclonal immunoglobulin D is found in the serum, so that the disease is also called Hyper-IgD Syndrome (HIDS), but this is neither specific, as it can also be found in some patients with other PFSs, nor sensitive, as younger patients may have normal IgD values.

In MKD, steroids are administered during febrile attacks, but for some patients with long-lasting flares, the treatment becomes continuative. Other treatments (colchicine, cyclosporine, thalidomide, and statins) are of little benefits and biological drugs (anakinra and etanercept) have been used with some success as steroid-sparing agents [24]. The heterogeneous results obtained by novel biologic treatments [27, 28] remain unclear since the molecular events leading to inflammatory phenotype are still unknown, and a role of cell apoptosis has been recently proposed in the MKD pathogenesis [29, 30].

### 2.3. Tumor-Necrosis-Factor- (TNF-) Receptor-Associated Periodic Syndrome (TRAPS)

TRAPS was first described in 1982 in a family of Irish and Scottish descent and was initially called “familial Hibernian fever” [31]. Since then, cases have been identified in other populations [32]. Mean age at onset is approximately 10 years. Each attack lasts few days to few weeks. Sometimes, in addition to fever, the attacks are characterized by severe abdominal pain, which may mimic a surgical emergency. Cutaneous manifestations are present in 87% of patients during attacks [33]. Most patients exhibit localized painful erythematous macules and patches that tend to migrate to the distal parts of the extremities.

High-dosage corticosteroids are administered during febrile attacks. Colchicine and immunosuppressant are ineffective. Etanercept, to prevent febrile attacks and to avoid long-term renal complications, is effective in a subgroup of patients, while IL-1*β* inhibitors are effective in most. On the contrary anti-TNF monoclonal antibodies (infliximab or adalimumab) have been shown to worsen the inflammatory condition [16].

### 2.4. NOD-Like-Receptor-Protein (NLRP-) Related Diseases

NLRP-related disease comprises phenotypically distinct autosomal dominant syndromes, such as NLRP3-associated Familial Cold Autoinflammatory Syndrome (also named FCAS1) and NLRP12 associated periodic fever (also named FCAS2).

#### 2.4.1. Familial Cold Autoinflammatory Syndromes-1 (FCAS1)

FCAS1, formerly known as familial cold urticaria, was first reported in 1940 [34], and, since then, only 20 families have been described worldwide. It is a rare autosomal dominant syndrome caused by a mutation in the NLRP3 gene, located on chromosome 1p44 that encodes a pyrin-like protein, known as cryopyrin [6]. There are three distinct clinical disorders related to NLRP3 mutations (FCAS1, MWS, and CINCA) that can now be seen as a single disorder with variable phenotypic expression [35–37]. 

FCAS1 is characterized by attacks of fever, urticarial skin rash, arthralgia, and conjunctivitis brought on by exposure to cold. An episode starts 2-3 hours after exposure and generally subsides within 24 hours. Attacks are accompanied by an intense acute phase response, as evidenced by high leukocyte counts in peripheral blood [38, 39].

In patients with FCAS1, treatment with IL-1*β* antagonists (i.e., anakinra) allows a complete response for almost all patients, but it has limited effects on hearing loss, bone dysplasia, and mental retardation [24].

#### 2.4.2. Familial Cold Autoinflammatory Syndrome-2 (FCAS2)

FCAS2 is a more recently characterized condition, firstly described in two families from Guadeloupe [37]. The disease is secondary to mutations in the *NLRP12* gene with an autosomal dominant inheritance. It was the first example of an NLRP capable of negatively regulating NF-*κ*B activation [40], and it could be classified as a NF-*κ*B activation disorder [41]. The phenotype is characterized by recurrent episodes of fever, secondary to cold exposure, associated with other signs, as skin rash, lymphadenopathy, aphthous ulcers, and abdominal pain [42]. A few patients, so far, have been presenting a good response to anti-inflammatory drugs, given at occurrence, while treatment with anakinra induced only an initial good response, that decreased over time [24].

### 2.5. Periodic Fever, Aphthous Stomatitis, Pharyngitis, and Cervical Adenitis (PFAPA)

PFAPA syndrome was firstly described in 1987 by Marshall et al. [43] and is characterized by episodes of fever, generally higher than 39°C, lasting for 3–6 days with recurrences every 3–8 weeks. Fever attack is associated with at least one of three main signs: aphthous stomatitis, cervical adenitis, and pharyngitis. The onset of the disease usually occurs before the age of 5 years and generally resolves by adolescence. Diagnosis is established on the basis of clinical criteria by which cardinal signs and symptoms must be carefully observed for a differential diagnosis [44, 45]. Periodic fever with tonsillitis can be part of the clinical phenotype also in some of the previous mentioned monogenic PFSs, even if these diagnoses should be considered only in the presence of atypical signs, such as clinical relapse after tonsillectomy, complaint of severe symptoms not referred to ear-nose and throat, and absent response to glucocorticoids [5].

Corticosteroids (prednisone, betamethasone) are successfully used in PFAPA syndrome during the febrile flares, as they can dramatically switch off fever in few hours. Other accompanying symptoms, however, take longer to resolve, and corticosteroid therapy sometimes can lead to shorten the interval between attacks. Colchicine, used as prevention of febrile attacks, can induce an increased interval between fever attacks but not a complete remission. Cimetidine, a histamine type 2 receptor blocker with immunomodulating properties, was shown to be effective in only one-third of patients [45]. Although the role of tonsillectomy is still controversial, it remains the most effective intervention for long-term resolution of PFAPA syndrome symptoms [44, 46].

## 3. Molecular Pathophysiology of PFSs

Recently, Masters et al. [41] proposed a classification scheme for the autoinflammatory disorder based on molecular mechanisms rather than on clinical classification. According to this scheme, PFSs were classified into six categories: (1) IL-1*β* activation disorders (inflammasomopathies), (2) nuclear factor (NF)-kappaB (NF-*κ*B) activation syndromes, (3) protein misfolding disorders, (4) complement regulatory diseases, (5) disturbances of cytokine signaling, (6) macrophage activation syndromes. The PFSs share the dysregulation of NLR and TLR family genes in the development of the diseases.

The inflammasomopathies include intrinsic inflammasomopathies (such as FCAS1 [47, 48]), extrinsic inflammasomopathies (such as FMF [49, 50] and MKD [51, 52]), and Complex/Acquired Inflammasomopathies. This category was characterized by an activation of NLRP3 (originally denoted cryopyrin or CIAS1) that processes pro-IL-1*β* into an active form, mature IL-1*β*. The difference between intrinsic and extrinsic forms depends on whether the mutation is on NLRP3 constituents complex or on proteins that regulate the production of IL-1*β*, respectively.

The NF-*κ*B activation syndromes are mediated predominantly by improper regulation of NF-*κ*B within the innate immune system (i.e., FCAS2). In NF-*κ*B/FCAS2, although clinically similar to FCAS1, the mutational screening in NLRP3 and other known periodic fever genes is negative [37]. A subsequent examination of NLRP12 (NALP12, PYPAF7, and MONARCH-1), chosen because of its similarity to NLRP3 and because of its expression in myelomonocytic cells, revealed dominantly inherited nonsense and splice-site mutations in the two families. Moreover, NLRP12 was the first example of an NLR protein capable of negatively regulating NF-*κ*B activation [40]. It is not known whether NLRP12 can participate in an inflammasome complex regulating IL-1*β* production.

The TRAPS syndrome instead is an example of a protein misfolding disorder. This kind of disorder is caused by multiple mechanisms leading to several problems, that can vary from misfolding of proteins to production of pro-inflammatory cytokines by innate immune cells [53].

Finally, there are a number of disorders that appear clearly to be autoinflammatory, but for which there are insufficient data to place them into one of the six categories. An example is the PFAPA, which is not inherited as a mendelian trait, but does exhibit some familial tendency [54, 55]. While the exact cellular determinants and pathogenic molecular mediators remain elusive, the clinical picture and cytokine profile of PFAPA outline a very complex, heterogeneous, but nevertheless autoinflammatory, disease [55].

## 4. Genetic Testing

Molecular analysis of periodic fevers' causative genes can dramatically improve patient quality of life by allowing early and accurate diagnosis and the administration of appropriate treatments. 

**Figure 1:**
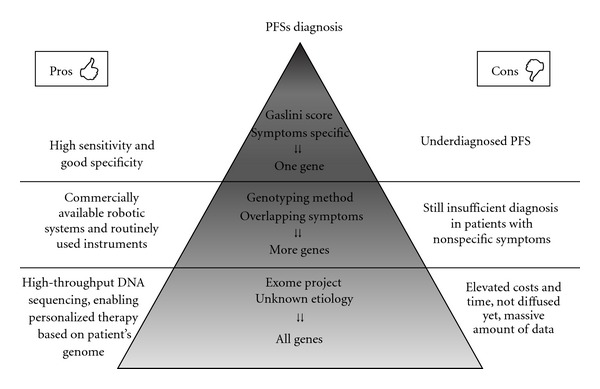
Schematic representation of the possible diagnostic methods for periodic fever syndromes.

However, the molecular genetic analysis of these diseases based solely on the candidate gene has low efficiency (close to 20%) and is time consuming and expensive. Moreover, since most of the causative mutations are located in specific regions of the genes mainly involved in these disorders, only some exons are analyzed for genetic diagnosis [56, 57] (Figure 1). The greatest difficulty is to decide which gene should be first screened, based on patient's clinical features. This is true in particular for patients with low penetrance mutations in whom the clinical manifestations of the disorders can show wide overlapping. Aimed at ameliorating the efficiency of the genetic diagnosis, a set of clinical parameters that can predict the probability of carrying mutations in one of the genes associated with hereditary autoinflammatory syndromes, has been proposed by Gattorno et al. [58]. In particular, the study allowed to identify some clinical variables (family history, age of onset, presence of abdominal, and chest pain, diarrhea) that are strongly associated with the probability of detecting relevant mutations in known PFS genes, thus also suggesting the order in which the genes should be screened. This diagnostic score revealed high sensitivity (87%) and good specificity (72%) for the identification of genetically positive and negative patients. Moreover, Federici et al. [59] performed a similar study in a large group of adult patients screened for three genes (*MEFV*, *MVK*, and *TNFRSF1A*) and obtained results comparable with the study carried out on children. 

Unfortunately, despite the application of this validated diagnostic system, literature data support the evidence that PFSs are underdiagnosed or not correctly classified. As reported by Lainka et al. [60], in Germany FMF is the most frequent PFS, but its incidence is low and, at present, genetic testing should be considered after an accurate clinical history including ethnic ancestry and family history [60, 61]. Besides, the overlap and the wide range of clinical signs shared by different PFSs and/or other diseases may cause a delayed or incorrect diagnosis [62]. In these cases, clinicians can be supported in a faster and correct identification by appropriate genetic tests.

An ordinary medium-throughput genotyping method based on a 96-well sequencing plate can also be developed to analyze the most frequently PFS causing genes by automating several processes required for direct sequencing, using a commercially available robotic systems and routinely used instruments [63]. This medium-throughput genotyping method may improve diagnostic success in patients with overlapping phenotype but cannot completely solve all the lacks of correct evaluation that still exist in PFS diagnostics.

High-throughput DNA sequencing (HTS) could be also used for sequencing patients' whole genomes or targeted regions such as all exonic regions (i.e., the exome) [64]. The objective is the identification of genetic variants such as single nucleotide polymorphisms (SNPs) that can influence the clinical phenotype. The extraction of SNPs from the raw genetic sequences involves many processing steps and the application of a diverse set of tools, and thus its use is mainly limited to research [65]. These second-generation sequencing technologies, including resequencing microarray, allow for multiple gene tests in a single experiment and thus hold considerable promise for routine molecular diagnosis of heterogeneous disorders [66]. Recent data confirmed the key role of this technology in the diagnosis of autoinflammatory diseases [67].

The expectation is that, in the very near future, this technology will enable us to identify all the variants in an individual's personal genome and, in particular, clinically relevant alleles. Beyond this, whole genome sequencing is also expected to bring a major shift in clinical practice in terms of diagnosis and understanding of diseases, ultimately enabling personalized medicine based on one's genome [68]. However, increasing the potential of genetics can bring to the attention new problems linked to the right interpretation of a huge amount of data.

## 5. Conclusions

PFSs are increasingly recognized in current clinical practice. This is probably due to a diminished clinical impact of infections at present times and to a wider awareness of the problem among physicians. PFAPA is by far the most common of PFSs and in most cases it can be easily diagnosed on the basis of the typical clinical presentation, usually in young children and with complaints mainly limited to pharynx, mouth, and neck. On the contrary, atypical PFAPA and other kinds of periodic fever can be a diagnostic challenge, even for experienced physicians. In some cases, the clinical picture may suggest a specific PFS, and the diagnosis can be confirmed on the basis of specific clinical features, response to drugs, and ultimately molecular analyses. However, patients may present incomplete phenotypes that can be compatible with different PFSs, making it difficult for the choice of the correct analysis to perform. In such cases, the analysis of a set of different genes, thanks to the availability of new technological platforms, can help the clinician. Near half of patients may remain without a genetic diagnosis even after performing multiple-gene analysis. In few of these cases, high-throughput genetic analysis will probably allow to identify novel monogenic disorders, but it is reasonable to hypothesize that a consistent percentage of patients will still remain without a definite diagnosis. The application of bioinformatics to high throughput genetic analysis could allow the identification of complex genotypes, but the complexity of this definition will hardly result in a clear contribution for the physician. 

In conclusion, great improvements have been obtained in recent years in the field of PFSs thanks to the identification of a genetic cause for different disorders, and novel knowledge, including novel doubts, will come from high-throughput molecular analysis. In our opinion, however, to obtain the best from these new developments, a rule should always be kept in mind: use genetics only to answer specific clinical questions. 

## Figures and Tables

**Table 1:** Genetic and clinical features of PFSs and correlated treatments.

Disease	*Gene * (Chromosome)	Transmission	Protein	Clinical manifestation	Treatment		
					Drugs	Biologics	Surgery
FMF	*MEVF* (16p13.3)	AR	Pyrin	Fever, serositis, sterile peritonitis, monoarthritis, pleuritis, and skin erythema	Colchicine	Anakinra Canakinumab Etanercept Infliximab Adalimumab	

MKD	*MVK* (12q24)	AR	Mevalonate kinase	Fever, lymphadenopathy, abdominal pain, and skin rash	Steroids	Etanercept Adalimumab Anakinra Canakinumab	

TRAPS	*TNFRSF1A* (12p13)	AD	p55 TNF-receptor	Prolonged fever, abdominal pain, erythematous macules, peritonitis, myalgias, arthralgias, periorbital oedema, and amyloidosis	High-dosage corticosteroids	Etanercept Infliximab Adalimumab Anakinra Tocilizumab	

NLRP-related diseases							
FCAS1	*NLRP3/CIAS1* (1q44)	AD	Cryopyrin	Fever, urticarial skin rash, arthralgia, and conjunctivitis		Anakinra Rilonacept, Canakinumab	
FCAS2	*NLRP12* (19q13)	AD	NLRP12	Fever, skin rash, lymphadenopathy, aphthous ulcers, and abdominal pain	Steroidal or nonsteroidal anti-inflammatory	Anakinra Rilonacept Canakinumab	

PFAPA	*ND *	ND	ND	Fever, pharyngitis, cervical adenitis, and aphthous stomatitis	Corticosteroids, colchicine		Tonsillectomy ± adenoidectomy

PFAPA: periodic fever, aphthous stomatitis, pharyngitis, and cervical adenitis; FMF: familial mediterranean fever; MKD: mevalonate kinase deficiency; NLRP: NOD-like receptor protein; CIAS: cold-induced autoinflammatory syndrome; FCAS: familial cold autoinflammatory syndrome; TRAPS: TNF-receptor-associated periodic syndrome; AR: autosomal recessive; AD: autosomal dominant; ND: not defined.
